# DISFIGURING MOLLUSCUM CONTAGIOSUM IN A HIV-POSITIVE PATIENT RESPONDING TO ANTIRETROVIRAL THERAPY

**DOI:** 10.4103/0019-5154.53188

**Published:** 2009

**Authors:** Sumit Sen, Bidyut Krishna Goswami, Nabendu Karjyi, Parna Bhaumik

**Affiliations:** *From the Department of Dermatology, Antiretroviral Therapy Centre, North Bengal Medical College and Hospital, India*; 1 *From the Department of Pathology, Antiretroviral Therapy Centre, North Bengal Medical College and Hospital, India*; 2 *From the Department of General Medicine, Antiretroviral Therapy Centre, North Bengal Medical College and Hospital, India*; 3 *From the Department of Medical Officer Antiretroviral Therapy Centre, North Bengal Medical College and Hospital, India*

**Keywords:** *Atypical molluscum*, *HIV*, *highly active antiretroviral therapy*

## Abstract

Molluscum contagiosum (MC) is caused by a double stranded DNA virus belonging to the pox virus family. MC lesions are usually pearly, dome shaped, small, discrete lesions with central umbilication. In HIV-positive patients atypical varieties are found. They may be large or nonumbilicated. Individual papules may join to form the agminate variety. This form is rare. Lesions of MC in healthy immunocompetent patients may occur at any part of the body including face, trunk, and limbs. Sexually active adults have lesions usually on the genitalia, pubis, and inner thigh, rarely on the face and scalp. We present a case of agminate MC occurring in a patient with acquired immunodeficiency disease responding to highly active antiretroviral therapy.

## Introduction

Lesions of MC are usually small papules 3-5mm in diameter with central depression and may spread along the line of trauma. Individual papules may grow to large size in AIDS patients and may become numerous.[1] The lesions rarely join to form a large plaque. MC in AIDS patients are resistant to conventional treatment used to treat those in immunocompetent patients. They may subside when the immunological status improves with antiretroviral therapy only, obviating the need for any local therapy. Reports of such cases are rare.

## Case Report

**Figure 1:**
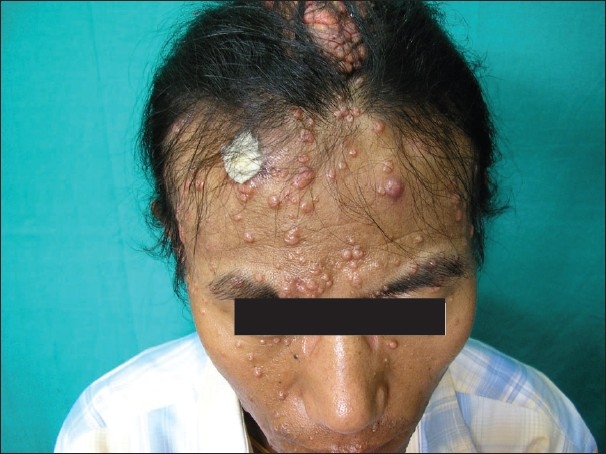
Disfiguring molluscum on face and scalp of HIV-positive patient

**Figure 2:**
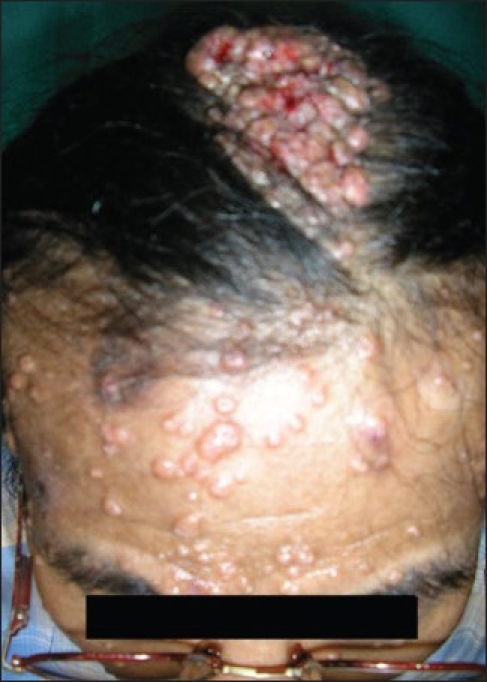
Numerous molluscum have joined to form large plaque on the scalp

**Figure 3:**
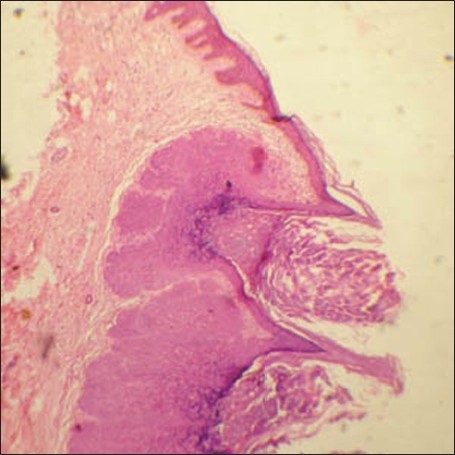
Molluscum bodies in the dermis (H&E, × 10)

**Figure 4:**
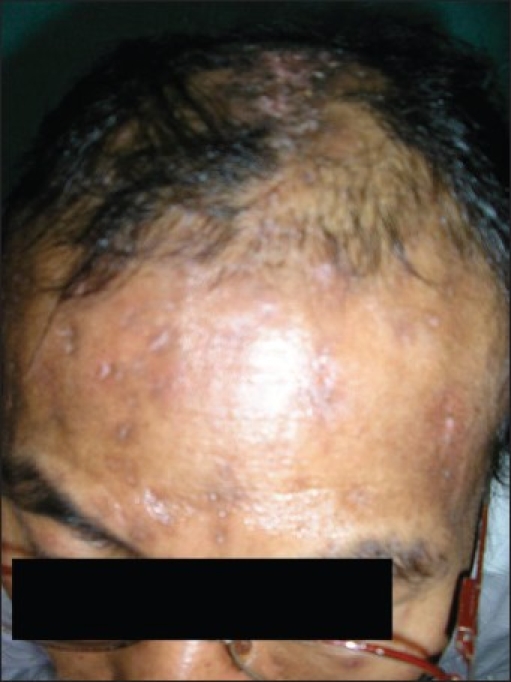
Molluscum contagiosum lesions have disappeared after six months of treatment

DS, a 48 year-old man, was referred to the Dermatology department from the Antiretroviral Therapy (ART) Centre. The patient had numerous waxy, small, nontender lesions on his face, neck, and scalp [Figure 1]. Lesions on the face and neck were 5 mm in diameter on average and numbered more than fifty. Numerous papules on the scalp had joined together to form an enlarged structure more than 3.5 inch in length producing a unique appearance [Figure 2]. Small lesions were scattered over the upper trunk, but there were no lesions over the genitalia. The person was unmarried and had the habit of frequenting female commercial sex workers for the last fifteen years. He gave no history of blood transfusions or drug abuse. Routine blood test revealed a leukocyte count of 13000/cm, and routine urine examination was normal as was the chest X-ray. The Venereal Disease Research Laboratory test for syphilis was nonreactive as was the HbsAg. He was detected to be HIV positive (HIV1) on two occasions and his CD4^+^ count was 58 cells/mm^3^ at this time. A biopsy was taken from one of the facial lesions and was stained with H&E stain. MC bodies were seen in the dermal skin [Figure 3]. He was placed on highly active antiretroviral therapy (HAART) with a regime of zidovudine 300mg twice daily, nevirapine 200mg twice daily, and lamivudine 150 mg twice daily. The patient was followed up and six months following the above regime the plaque lesion on the scalp and lesions on the face had resolved completely leaving scars [Figure 4]. His CD4^+^ count at this time had risen to 226 cells/mm^3^. Five months after complete resolution of the lesions there is no sign of any relapse of the molluscum.

## Discussion

Molluscum contagiosum lesions are seen in 10-20% of AIDS patients.[1] They are usually flesh coloured, numerous, occurring around the genital region. Infective lesions may rarely occur on the face and may occur in large numbers in men in this region due to shaving. They remain discrete. Atypical clinical varieties such as the giant molluscum, molluscum presenting as an abscess, molluscum without any umbilication, tender molluscum, and erythematous nodular types have been reported in AIDS patients.

The lesions of MC that occur in AIDS patients differ in size, site, and morphology from those occurring in the immunocompetent. Lesions in the symptomatic HIV-positive patients however usually remain separate from one another and rarely conglomerate to form a large plaque known as the agminate type.[2]

The transmission of MC is by autoinoculation and close physical contact. Anecdotally, wrestlers have been recorded to transmit this infection from one to another.[3] Spread is also possible by fomites.

Diagnosis is best established by biopsy staining of the curdy material with Giemsa and demonstration of eosinophilic molluscum bodies. Atypical histopathological varieties are seen in these patients. Verrucous and hyperkeratotic varieties may occur.

In immunocompetent healthy individuals the lesions usually last 6-12 months. Persons with AIDS have papules which are often resistant to conventional modalities of therapy like curettage, electrocautery, thermal cautery with trichloroacetic acid, cryotherapy, and topical imiquimod.[4] Intralesional interferon used in recalcitrant cases too have failed. Lesions are known to recur after initial response in these patients. A direct correlation exists between the degree of immunosuppression and response to therapy of MC in AIDS. Antiretroviral therapy with various combinations of antiretroviral drugs has been reported to lead to a complete and stable resolution of lesions of MC by improving the immune status of the patient. Hurni *et al.*, described complete disappearance of MC in HIV patients using saquinavir, zidovudine, and lamivudine.[5] A combination of indinavir, lamivudine, and stavudine in another case has been reported to produce the desired result.[6] Indinavir in combination with two reverse transcriptase inhibitors has resulted in resolving the lesions of three resistant cases of MC in HIV.[7] We used a different combination in our regime. HAART has been shown to completely cure a case of conjunctival MC in an AIDS patient.[8] Ritonavir, another antiretroviral drug, has been known to resolve lesions of MC in AIDS patient.[9] The immune response inflammatory syndrome (IRIS) is an inflammatory state which occurs in AIDS patients and where the patient's condition clinically worsens after institution of HAART. New lesions often arise. Persistence with HAART has been reported to be beneficial in MC in AIDS patient suffering from IRIS.[10]

We discuss this rare case of disfiguring and multiple MC responding rapidly to antiretroviral therapy, as it suggests the exciting possibility of HAART as the treatment of choice for all cases of molluscum in AIDS patients without the need for any topical interference.
